# Host identity dominates over intra-ruminal spatial variation in shaping the rumen microbiome of Holstein–Friesian dairy cows

**DOI:** 10.3389/frmbi.2026.1883079

**Published:** 2026-08-31

**Authors:** Soumya Kanti Kar, Edoardo Zaccaria, Léon Šebek, Sanne van Gastelen

**Affiliations:** Department of Animal Nutrition, Wageningen Livestock Research, Wageningen, Netherlands

**Keywords:** 16S rRNA gene sequencing, dairy cattle, Holstein–Friesian, rumen cannulation method, rumen microbiome, spatial variation

## Abstract

The rumen is a structurally complex fermentation chamber with distinct physicochemical zones, resulting in intra-ruminal biogeography and raising the question whether microbial communities vary spatially within the rumen. We investigated the extent of intra-ruminal microbial variation by analyzing rumen fluid collected from six rumen cannulated Holstein–Friesian dairy cows. Rumen fluid samples were obtained in duplicate from three anatomical rumen regions: the cranial sac (Front), the front ventral sac (Middle), and the middle ventral sac (Back).and subjected to 16S rRNA gene amplicon sequencing. Hierarchical clustering, ordination, and redundancy analyses revealed a strong host-specific signature, with individual cow explaining 37 to 43% of the total community variation (p = 0.001). In contrast, neither rumen sampling location nor technical duplicate significantly affected alpha- or beta-diversity metrics. Only a small subset of low-abundance taxa (<1% of the total community) differed between the rumen sampling locations, whereas core genera such as *Prevotella*, *Christensenellaceae* R-7 group, *Lachnospiraceae* NK3A20 group, *Rikenellaceae* RC9 gut group, and *Methanobrevibacter* were consistently dominant across sites. Differential abundance analysis revealed significantly different ASVs across rumen sampling locations, despite no major shifts in overall community structure. These results demonstrate that the rumen microbiome is stable within individual cows with negligible spatial structuring across the horizontal plane, although caution is warranted before extrapolating this uniformity to vertical stratification layers or alternative sampling techniques.

## 1 Introduction

The rumen functions as a specialized anaerobic fermentation chamber where diverse microbial consortia including bacteria, archaea, protozoa, and fungi degrade fibrous plant material into volatile fatty acids (VFA) and other metabolites that serve as the primary energy source for the host (Hagey et al., 2022; Soltis et al., 2023a). The interior of the rumen is structurally complex, comprising sacs separated by muscular folds and pillars. This physical form of the rumen not only increases surface area but also establishes distinct physicochemical microenvironments, such as the stratification of digesta into a fibrous mat overlaying a liquid phase (Shen et al., 2012; Mizrahi and Jami, 2018). As a result, gradients in pH, VFA concentrations, and redox conditions can emerge across regions, creating biogeographically distinct microbial habitats within the rumen (Duffield et al., 2004; Shen et al., 2012; Soltis et al., 2023a).

Although these spatial gradients are well documented at the physiological and chemical level, the majority of rumen microbiome studies still rely on samples collected from a single site. Several collection methods are available, with the rumen cannulation method (RC) and the oral stomach tube method (OST) being most frequently used (Duffield et al., 2004). The OST method allows rumen fluid to be obtained through the esophagus using intact animals, and is considered to be less invasive than the RC method. This makes this method applicable to large-scale studies. However, the OST method primarily recovers liquid digesta, with little to no representation of the solid (fiber-adherent) fraction where many key fibrolytic microbes reside (De Assis Lage et al., 2020; Hagey et al., 2022). Furthermore, OST samples generally only reflect the cranial dorsal or central side of the rumen and are vulnerable to saliva contamination, which can elevate pH and dilute metabolite concentrations (Mizrahi and Jami, 2018; Hagey et al., 2022). These limitations raise concerns about how representative OST-derived microbial profiles are of the whole rumen ecosystem.

In contrast, with the RC method, rumen fluid is collected through the fistula from rumen cannulated animals. This method is widely regarded as the gold standard for microbiome studies for number of reasons. First, the RC method provides direct access to the reticulo-rumen (De Assis Lage et al., 2020). Second, grab samples that include both solid and liquid fractions can be collected using the RC method, providing a more accurate representation of the microbial community involved in fiber degradation. Third, the RC method allows targeted sampling of defined anatomical sites, thereby reducing sampling bias and capturing biogeographical variation within the rumen. Fourth, the risk of saliva contamination is considered to be absent with the RC method (Hagey et al., 2022).

Despite the fact that the RC method is uniquely suited for investigating spatial variation in rumen microbial ecology, this has not been done yet in Holstein-Friesian (HF) dairy cows. At present, only Soltis et al. (2023a) explored the microbial community of five biogeographical regions in the rumen of rumen cannulated Angus cows (beef cattle), and concluded that within the bacterial community neither alpha- nor beta-diversity differed, but that relative abundance was affected. Therefore, the aim of the study was to investigate the spatial variation in microbial diversity and community across three anatomical sites in the rumen using RC HF dairy cows. We hypothesized that physical stratification within the rumen would result in niche-specific differences in the relative abundance of bacterial taxa across the three anatomical sampling locations, while overall alpha- and beta-diversity would remain largely uniform. This hypothesis was evaluated by comparing microbial communities among three horizontally distributed rumen sites.

## 2 Materials and methods

### 2.1 Animal, experimental design and sampling

The rumen fluid samples used in this study were collected from dairy cows enrolled in an associated experiment previously described by (van Gastelen et al., 2021). The aim of that experiment was to induce hindgut and metabolic acidosis via abomasal infusion of corn starch and β-hydroxybutyrate (BHB), respectively, and to assess their effects on performance and metabolism. The present study included only rumen samples collected during the water-control infusion period. Ruminal pH and volatile fatty acid (VFA) measurements from the parent experiment have been reported previously (van Gastelen et al., 2021) and were therefore not repeated here, as the focus of this study was the rumen microbiome. The experiment was conducted in 2019 at the animal research facilities of Wageningen University & Research (Wageningen, the Netherlands), under the Dutch Law on Animal Experiments and in accordance with European Union Directive 2010/63. It was approved by the Central Committee of Animal Experiments (The Hague, the Netherlands; 2018.D-0013.002) and the Animal Welfare Body of Wageningen University.

Six rumen-cannulated, multiparous HF cows producing 28.8 ± 4.96 kg/d at 66 ± 18 DIM (average ± SD) were randomly assigned to a 6 × 6 Latin square design with six treatments. Cows were adapted to the experimental conditions for 19 d before the first period. Each experimental period (n = 6) lasted 7 d, consisting of 5 d of continuous abomasal infusion followed by 2 d of rest. During this time, cows were housed individually in identical climate respiration chambers and fed a restricted (90% of voluntary intake) total mixed ration of 35.0% grass silage, 37.4% corn silage, and 27.6% concentrate (dry matter basis). Feed was delivered in equal portions every two hours via an automated system to promote metabolic steady-state conditions, and cows had free access to water.

**Figure 1:**
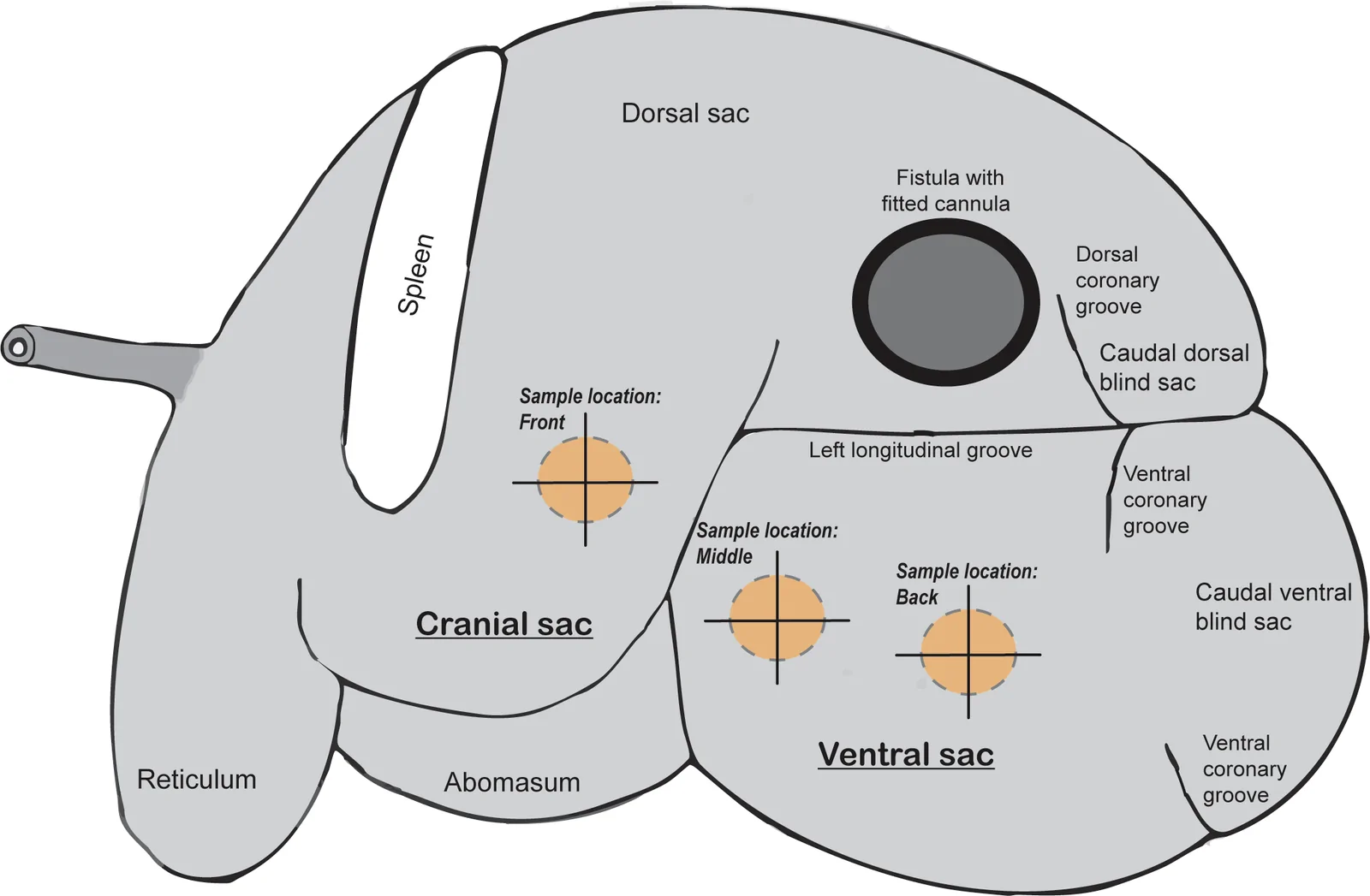
Sampling of rumen fluid in rumen-cannulated cows. Left lateral view of the rumen showing a fistula with cannula at the left paralumbar fossa. Samples were collected from three regions: *Sample location: Front* (cranial sac), Sample location: Middle (front ventral sac), and Sample location: Back (middle ventral sac).

On the final day of the experimental period, rumen fluid samples were collected from each cow in technical duplicates, i.e. primary and duplicate samples. Sampling targeted three distinct rumen locations: the cranial sac, front ventral sac and the middle ventral sac, following the procedure of van Zijderveld et al. (2011), without pooling. Each site was sampled separately using dedicated equipment consisting of a perforated polyvinyl chloride tube and vacuum hand pump. This approach ensured that rumen fluid from the three locations remained distinct, allowing site-specific characterization (**Figure 1**). Samples were collected from three regions in the rumen, i.e.: cranial sac for sample location: Front; front ventral sac for sample location: Middle; and middle ventral sac for sample location: Back. Immediately after collection, fluid was sub-sampled into 12 mL tubes, placed on dry ice, and stored at −80 °C. For the present study, only rumen fluid samples from cows that received the control abomasal infusion treatments were used. This resulted in a total of 36 rumen samples collected from six individual fistulated cows (n=6). The samples were equally distributed across three sampling locations (front, middle, back; n=12 per location) and included technical duplicates.

### 2.2 DNA extraction, and library prep

Microbial DNA was extracted from the rumen fluid samples, and the hypervariable V4 region of the 16S rRNA gene was amplified and sequenced on an Illumina HiSeq platform at Genotypic Technology Pvt. Ltd. (Bangalore, India). Detailed protocols are available in Roques et al. (2023). Briefly, DNA was extracted with the Qiagen DNeasy Blood & Tissue Kit (Qiagen, Hilden, Germany) and quality-checked by agarose gel electrophoresis. The V4 region of the 16S rRNA gene (Caporaso et al., 2011) was amplified with primers 515F (GTGCCAGCMGCCGCGGTA) and 806R (GACTACHVGGGTATCTAATCC) using a two-step PCR, and amplicons were sequenced on an Illumina HiSeq X Ten platform (2 × 150 bp, ≥0.7 M paired-end reads/sample).

### 2.3 Bioinformatics and statistics

Primer trimming was performed using cutadapt; denoising, merging of paired reads, and chimera removal were carried out using DADA2 version 2023.9.0 (Callahan et al., 2016) to infer amplicon sequence variants (ASVs). Taxonomy was assigned with a Naive Bayes classifier trained on SILVA v138.2. Non-target ASVs (chloroplast/mitochondria) were discarded. All groups (i.e., 6 cows, 3 sample locations within the rumen, and 2 technical duplicates) yielded a comparable sequencing depth, with most samples yielding between 2.0×10^5^ and 4.5×10^5^ reads (**Supplementary Figure 1**). Bacterial features were filtered using the following criteria: (1) present in more than 5% of samples and (2) a mean relative abundance greater than 0.01. After performing quality control (i.e., removal of chloroplast- and mitochondria-associated ASVs, confirmation of comparable sequencing depth across samples, and filtering of low-prevalence and low-abundance bacterial features), the final dataset comprised 1,404 ASVs and approximately 11.17 million high-quality reads. This process retained ∼88% of the initial reads, resulting in a mean of ∼310,000 high-quality reads per sample.

Data analyses were performed in R using phyloseq (McMurdie and Holmes, 2013), vegan (Oksanen et al., 2001), and DESeq2 (Love et al., 2014). Relative abundances were transformed as log10 (10,000 × relative abundance + 1). Samples were hierarchically clustered using Bray–Curtis dissimilarities computed on relative-abundance ASV profiles, with agglomerative average-linkage (UPGMA) implemented via hclust (method = “average”). Beta-diversity was assessed using Bray-Curtis dissimilarities calculated from relative-abundance ASV profiles. Community structure was visualized by principal coordinate analysis (PCoA), and differences in community composition were tested using PERMANOVA (adonis2, 999 permutations). The model included technical duplicate and rumen sampling location as fixed terms, with permutations restricted within cow to account for repeated measurements from the same animal. To evaluate whether the inclusion of technical duplicates influenced the results, the PERMANOVA was repeated using only the primary sample from each cow and sampling location. Homogeneity of multivariate dispersion among sampling locations and technical duplicate groups was assessed using PERMDISP (betadisper followed by permutest, 999 permutations). Redundancy analysis (RDA) was performed on log-transformed relative abundance matrices to evaluate the influence of rumen location, cow, and technical duplicate on community composition. In partial RDA models, cow and technical duplicate were included as conditioning variables, with rumen sampling location specified as the explanatory variable. Alpha-diversity (Observed richness, Shannon, and Simpson indices) was calculated on rarefied count tables (rarefaction depth: 106,840 reads per sample, corresponding to the minimum post-QC depth). Differences between rumen locations were evaluated using linear mixed models (lmer), with rumen location and technical duplicate as fixed effects and animal included as a random effect. Differential abundance analysis was performed at the ASV level using DESeq2 with the design formula ~ Cow + TechnicalDuplicate + Location. Pairwise contrasts were tested between the three rumen sampling locations. P-values were adjusted for multiple testing using the Benjamini-Hochberg false discovery rate (FDR), and ASVs with FDR < 0.05 were considered significant. For significant ASVs, taxonomic assignment, log2 fold-change, adjusted p-value, mean relative abundance per location, and prevalence across samples were reported in **Supplementary Table 1**. As an exploratory functional prediction, PICRUSt2-derived pathway, KO, and EC abundance profiles were analyzed using Bray-Curtis dissimilarities and PERMANOVA with permutations restricted within cow.

## 3 Results

**Figure 2:**
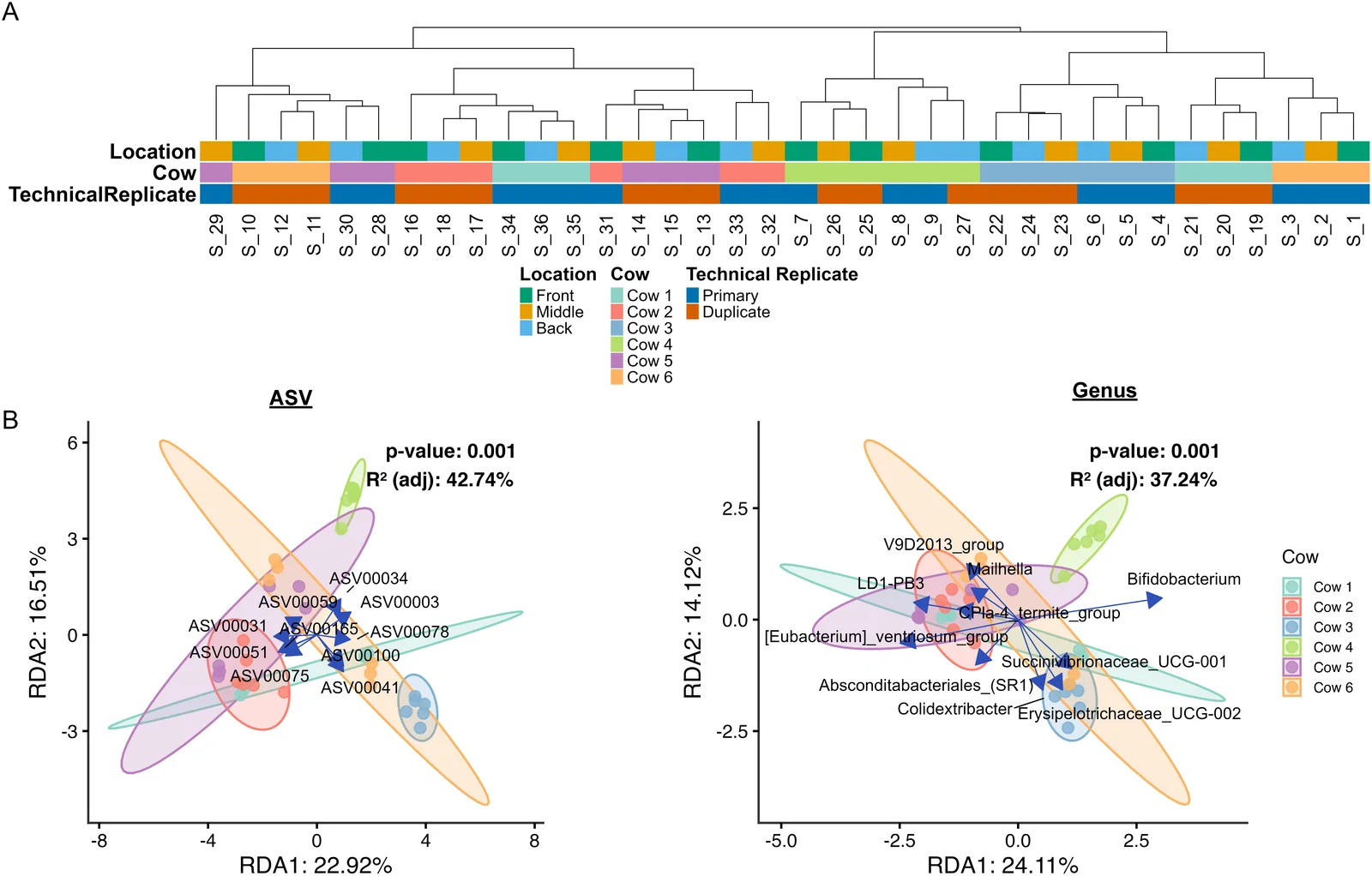
Individual-level clustering and ordination of rumen microbial communities in rumen-cannulated Holstein–Friesian cows. **(A)** Hierarchical clustering of rumen microbiome profiles based on Bray–Curtis dissimilarity. Colored bars beneath the dendrogram denote technical duplicate (primary or duplicate), cow, and rumen sampling location (front, middle, or back). **(B)** Redundancy analysis (RDA) illustrating microbial community separation by cow. Each ellipse represents the 95% confidence interval around samples from the same cow. Arrows indicate amplicon sequence variants (ASVs, bottom left) or genera (bottom right) contributing most strongly to the ordination axes. The percentages on the axes denote the proportion of total variance explained. Significance was assessed using permutation tests (p = 0.001).

Hierarchical clustering based on Bray-Curtis dissimilarity revealed a clear host-specific pattern in the rumen microbiome composition (**Figure 2A**). Samples from the same cow clustered together regardless of rumen location (front, middle, or back) or technical duplicate (primary or duplicate). Consistently, RDA demonstrated clear separation by cow (p = 0.001; **Figure 2B**), with individual cow explaining 37 to 43% of the total variation. PCOA (**Supplementary Figure 2A**) showed overlapping community profiles between the different rumen sampling locations and with pRDA no significant separation was observed between the different rumen sampling locations (adjusted p = 0.92, R² < 0.001; **Supplementary Figure 2B**). PERMANOVA showed no detectable effect of technical duplicate (R² = 0.023, p = 0.259) or rumen sampling location (R² = 0.027, p = 0.810). Homogeneity of multivariate dispersion was confirmed for both rumen sampling location (PERMDISP, p = 0.600) and technical duplicate (PERMDISP, p = 0.518), indicating that the PERMANOVA results were not driven by unequal within-group dispersion. In the sensitivity analysis using only primary samples, rumen sampling location remained non-significant (R² = 0.070, p = 0.144). These results demonstrate that inter-animal variation, and not intra rumen and technical variation, was the dominant driver of rumen microbiome composition. To assess whether local rumen micro-environments affected within-sample diversity, we compared alpha-diversity metrics (Observed, Shannon, and Simpson indices) after rarefaction to 106,840 reads per sample. No statistical significant differences were detected between the different rumen sampling locations (**Supplementary Figure 2C**), even when accounting for technical duplicate and cow. Together, with the beta-diversity analyses, these findings confirm that local rumen micro-environments exert minimal influence on microbial diversity and community structure, supporting the strong host-specific signature of the rumen microbiome.

**Figure 3:**
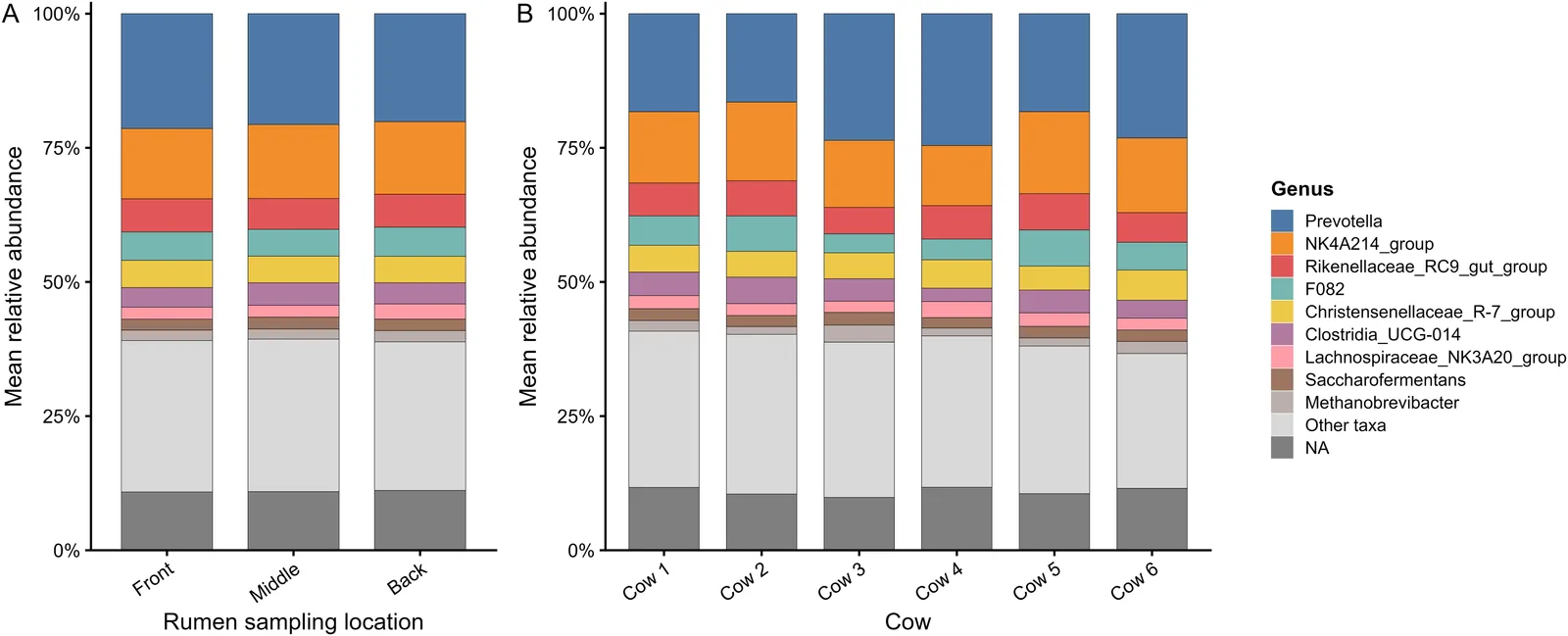
Genus-level rumen microbial composition across sampling locations and cows. Stacked barplots show the mean relative abundance of the 10 most abundant genus-level taxa, with all remaining taxa collapsed as “Other taxa”. **(A)** Mean genus-level composition across rumen sampling locations: Front, Middle, and Back. **(B)** Mean genus-level composition across individual cows. Relative abundances were calculated at the sample level before averaging within each group.

Differential abundance analysis identified 48 significant ASV-contrast associations across the three pairwise location comparisons, corresponding to 26 unique ASVs (**Supplementary Table 1**). These ASVs were generally low-prevalence and low-abundance features, with a median prevalence of 12.5% of samples and a median mean relative abundance of approximately 0.015%. Therefore, although some ASVs differed significantly between locations, these differences were restricted to sparse, low-abundance taxa and did not correspond to detectable shifts in overall community structure. (**Supplementary Figure 3**). The dominant genera included *Prevotella* (22%), *Christensenellaceae_R-7_group* (5.9%), *Rikenellaceae_RC9_gut_group* (6.6%), *Lachnospiraceae_NK3A20_group* (2.7%), and *Methanobrevibacter* (2.1%). Although some additional groups (e.g., NK4A214_group and F082) showed relatively higher abundances, they were not considered further due to their limited taxonomic resolution. Notably, *Methanobrevibacter* was the most abundant archaeal genus and ranked among the top 10 microbial genera overall (**Figure 3**).

As an exploratory functional prediction, PICRUSt2-derived pathway profiles were analyzed using Bray-Curtis dissimilarities and PERMANOVA with permutations restricted within cow. Consistent with the taxonomic analyses, predicted pathway profiles did not show a detectable effect of rumen sampling location (**Supplementary Figure 4**, R² = 0.048, p = 0.242). Similar results were obtained for predicted KO and EC profiles (KO: R² = 0.038, p = 0.401; EC: R² = 0.033, p = 0.505). These observations indicate that, although local microenvironments may slightly affect low-abundance ASVs, they do not substantially influence the overall community, and subtle quantitative differences between individual cows likely reflect host-specific modulation. Together, the ASV- and genus-level analyses support that host identity is the primary determinant of rumen microbial structure, with minimal impact from spatial variation within the rumen.

## 4 Discussion

Our analysis provides evidence that individual host differences shape the rumen microbiome, and that horizontal sampling location had no detectable effect on liquid-phase rumen microbial composition within the present experimental design. Samples from the same cow clustered tightly in hierarchical clustering and PCOA, regardless of whether they were collected from the front, middle, or back of the rumen, or were technical duplicates. Redundancy analysis confirmed that cow explained approximately 37 to 43% of the variance, whereas rumen sampling location had no effect on community composition. The variance not explained by cow may reflect residual biological heterogeneity, unmeasured temporal or microenvironmental factors, and technical or stochastic variation, which were not separately quantifiable in this study. This strong host-specific effect is consistent with previous work showing that individual animal factors (age, breed, diet, genetics) are major drivers of rumen microbial communities (Bowen et al., 2018). Recent metagenomic studies have also demonstrated that large fractions of the rumen microbiome are under host genomic control (Martínez-Álvaro et al., 2022). Thus, our findings demonstrate that the microbiota is primarily determined by host identity and that genetic and physiological differences between cows dominate over local rumen micro-environments.

Our hypothesis anticipated that physical stratification within the rumen would result in niche-specific differences in the relative abundance of bacterial taxa across the three sampling locations. While differential abundance analysis identified a limited number of significantly different ASVs, these differences did not translate into distinct community-level taxonomic profiles. Thus, our findings provide only partial support for the hypothesis, suggesting that under the present sampling strategy, horizontal spatial variation within the rumen is limited. This result aligns with Soltis et al. (2023b), who reported no differences in bacterial alpha- or beta-diversity across five anatomical rumen sacs in cannulated Angus cows. Although it has been suggested that sampling location within the rumen can influence microbial profiles (Dunière et al., 2025), due to stratification of digesta or pH gradients, our data do not support a meaningful effect of location.

Only subtle differences were detected at ASV level, differed significantly between locations, these differences were restricted to sparse, low-abundance taxa and did not correspond to detectable shifts in overall community structure. The consistent presence and relative abundance of “core taxa”, such as *Prevotella*, *Christensenellaceae* R-7 *group*, *Lachnospiraceae* NK3A20 *group*, *Rikenellaceae* RC9 gut *group*, and *Methanobrevibacter*, across all cows and rumen sampling locations suggest that these taxa are well conserved across HF dairy cows. *Prevotella* remained ones of the dominant genera under all conditions, in line with previous rumen studies (Henderson et al., 2015; Bowen et al., 2018), and likely contributes to the degradation of proteins and non-cellulosic polysaccharides during rumen fermentation. The other core taxa may support complementary fermentation processes and hydrogen turnover, although their precise functional roles require further validation. As an exploratory functional prediction, PICRUSt2-derived pathway profiles were also analyzed and showed no detectable effect of rumen sampling location, consistent with the taxonomic analyses. However, because these functional profiles were inferred from 16S rRNA gene data, they should be interpreted as exploratory support rather than direct evidence of functional equivalence. Shotgun metagenomics, metatranscriptomics, or metabolomics would be required to confirm whether functional potential or activity is truly conserved among rumen locations.

Importantly, technical duplicate had no detectable impact on microbial profiles, indicating high reproducibility of our sampling and sequencing methods. This suggests, together with the consistency observed across the sampled intra-ruminal sites, that a single well-collected rumen fluid sample per cow suffices to capture the microbial community within that specific ruminal plane. From a practical perspective, our findings simplify rumen microbiome studies utilizing the rumen cannula (RC) method: there is no apparent need to sample multiple horizontal intra-ruminal sites, which can be logistically challenging and resource-intensive.

However, these findings should be interpreted with caution regarding vertical stratification and alternative sampling techniques. While horizontal variation was negligible in this study, the rumen is characterized by distinct vertical layers, ranging from the floating solid mat to the ventral liquid phase, which harbor differing microbial communities (De Mulder et al., 2016). However, these results should not be simply extrapolated to the oro-stomach tube (OST) method. While our data indicates that the cranial sac (**Figure 1**) yields results representative of the horizontal rumen environment, the OST method introduces unique variables. The literature comparing RC and OST remains divided. Several studies that compared the RC and OST method, reported no differences in the microbiome community (Lodge-Ivey et al., 2009; Terré et al., 2013; Da Cunha et al., 2023), a substantial number of studies have identified clear differences between the two (Paz et al., 2016; Hagey et al., 2022; Pathak et al., 2025). These discrepancies likely stem from factors we cannot rule out in the OST method, such as sampling from more dorsal locations, the recovery of the liquid fraction only, and the potential for salivary dilution and contamination.

Despite the clear host-specific patterns observed, this study has some limitations. Rumen samples were obtained from a larger experiment involving abomasal infusions. Although the abomasal route of administration and the exclusion of treated samples minimize the likelihood of direct treatment effects, residual or indirect carry-over effects, if any, on rumen microbiota cannot be fully excluded and represent a limitation of the present study. The analysis was restricted to six HF cows under uniform management, limiting broader generalization. For example, the modest sample size of six cows, which may reduce power to detect small but biologically relevant location effects. Accordingly, the absence of significant differences in diversity metrics and ordination-based analyses should not be equated with absence of effect. Instead, our data indicate that any horizontal location effect in the sampled rumen plane was small relative to host identity and was mainly restricted to low-abundance taxa. Future studies with larger animal numbers, explicit power calculations, and estimation of confidence intervals around effect sizes and variance explained will be useful to refine these conclusions. An additional limitation is that only rumen samples were analyzed, without distinguishing between fiber-adherent (solid) and planktonic (liquid) fractions, leaving potential spatial variation in epithelium-associated (epimural) or particulate-associated microbiota unexplored. Dorsal–ventral stratification within the rumen was also not assessed and may reveal patterns not captured by our horizontal sampling scheme. Additionally, 16S rRNA profiling captures taxonomic but not functional variation, and sampling at a single time point does not account for temporal dynamics. Furthermore, analytical approaches like network analysis could be informative for exploring potential microbial associations in the rumen. However, we did not include such an analysis here because the limited number of animals, repeated sampling within cows, and the relatively small number of sampling locations reduce the robustness of network inference. Moreover, amplicon-based co-occurrence networks are vulnerable to compositional and sparsity-related artifacts. For these reasons, we focused on analyses directly relevant to our primary question, while noting that larger studies with complementary multi-omics approaches would be needed to robustly assess rumen microbial interaction networks. Nonetheless, our results provide a robust foundation for understanding host-driven rumen microbiome structure within the horizontal plane and liquid fraction sampled here, and for guiding efficient sampling strategies under comparable conditions.

In conclusion, these results provide a robust foundation for understanding host-driven rumen microbiome structure within the horizontal plane and liquid fraction sampled here, and for guiding efficient sampling strategies under comparable conditions. The predominant pattern is individuality: each cow possesses a distinct and stable microbial community, regardless of the sampling location within the rumen. These findings confirm and extend previous suggestions that host-specific factors outweigh rumen biogeography (Soltis, et al., 2023; Dunière et al., 2025). Together with recent studies on host-genome–microbiome interactions, rumen spatial ecology, and functional metagenomics (Dhillon et al., 2026), our findings suggest that future work should integrate host and site-specific factors to more fully resolve the mechanisms shaping rumen microbial assembly. By emphasizing the strength of the host effect, our work has important implications for rumen sampling methodology and for studies of host–microbiome interactions in dairy cattle.

## Data Availability

The datasets presented in this study can be found in online repositories. The names of the repository/repositories and accession number(s) can be found below: https://www.ebi.ac.uk/ena, PRJEB112917.

## Supplementary material

Supplementary Figure 1 Distribution of sequencing reads from rumen microbiome 16S rRNA gene sequencing across samples from 6 dairy cows **(A)**, 3 sampling locations in the rumen **(B)**, and technical duplicate **(C)**. The boxplots show the median, interquartile range, and 1.5× interquartile range, with individual points representing samples.

Supplementary Figure 2 Community structure and diversity across rumen locations. **(A)** Principal coordinate analysis (PCoA) at ASV level, points are colored by location (front, middle, back) and shaped by cow. **(B)** The pRDA of ASV log-centered relative abundances, constrained by rumen sampling location and conditioned on cow and technical duplicate. Axis labels show constrained variance (RDA1 = 0.66%, RDA2 = 0.60%); permutation test for rumen sampling location: p = 0.99; adjusted R² < 0.001. **(C)** Shannon diversity (ASV level) after rarefaction to 106,840 reads per sample, shown by rumen sampling location with points shaped by cow; no significant differences between the locations (mixed model, lmer). All panels use the same color/shape coding; n = 36 samples (12 per location).

Supplementary Figure 3 Relative abundance of the top 10 ASVs identified as significantly different across rumen locations (front, middle, back) by DESeq2 (FDR < 0.05). Boxplots show the distribution of relative abundances (y-axis) across the three rumen sampling locations, with individual points indicating samples and shapes denoting cows. Horizontal bars and asterisks denote significant pairwise contrasts (FDR corrected p-value < 0.05, ** < 0.01, *** < 0.001). Together, these ASVs accounted for a small fraction of the community (median ~0.6% of total abundance), underscoring that location effects were restricted to low-abundance taxa.

Supplementary Figure 4 Predicted functional pathway profiles across rumen sampling locations. Principal coordinate analysis (PCoA) based on Bray-Curtis dissimilarities of PICRUSt2-predicted pathway abundance profiles from rumen fluid samples collected from three rumen locations: Front, Middle, and Back. Points represent individual samples (n = 36), colors indicate sampling location, and shapes indicate cow identity (n = 6). Ellipses show the 80% confidence region for each sampling location. The first two PCoA axes explained 38.8% and 20.8% of the variation, respectively. Predicted pathway profiles showed substantial overlap among rumen locations, and PERMANOVA with permutations restricted within cow detected no significant effect of sampling location (R² = 0.048, p = 0.242). Because pathway profiles were inferred from 16S rRNA gene data, these results should be interpreted as exploratory functional predictions rather than direct metagenomic evidence.

Supplementary Table 1 Differentially abundant ASVs across rumen sampling locations. ASV-level differential abundance analysis was performed with DESeq2 using the model ~ Cow + TechnicalReplicate + Location. Pairwise contrasts were tested between the three rumen sampling locations: Front, Middle, and Back. The table reports significant ASV-contrast associations only (FDR-adjusted p-value < 0.05), including ASV identifier, log2 fold-change, adjusted p-value, taxonomic assignment, mean relative abundance, and prevalence across samples. Significant ASVs were generally low-prevalence and low-abundance features, and should therefore be interpreted as subtle location-associated differences rather than major shifts in community structure.
