# Supplementary material for: Host identity dominates over intra-ruminal spatial variation in shaping the rumen microbiome of Holstein–Friesian dairy cows
Source: Front Microbiomes. 2026 Aug 31;5:1883079. doi: 10.3389/frmbi.2026.1883079 (PMC13572667; doi:10.3389/frmbi.2026.1883079)
Supplement: Supplementary file 1 [file Supplementaryfile1.docx]

Supplementary Material

**Supplementary Figure S1: Distribution of sequencing reads from rumen microbiome 16S rRNA gene sequencing across samples from 6 dairy cows (A), 3 sampling locations in the rumen (B), and technical replicate (C).** The boxplots show the median, interquartile range, and 1.5× interquartile range, with individual points representing samples.

Supplementary Figure S2: Community structure and diversity across rumen locations. (A) Principal component analysis (PCA) at ASV level, points are colored by location (front, middle, back) and shaped by cow. (B) The pRDA of ASV log-centered relative abundances, constrained by rumen sampling location and conditioned on cow and technical duplicate. Axis labels show constrained variance (RDA1 = 0.66%, RDA2 = 0.60%); permutation test for rumen sampling location: p = 0.99; adjusted R² = 0.000. (C) Shannon diversity (ASV level) after rarefaction to 106,840 reads per sample, shown by rumen sampling location with points shaped by cow; no significant differences between the locations (mixed model, lmer). All panels use the same color/shape coding; n = 36 samples (12 per location).

**Supplementary Figure S3**. **Relative abundance of the top 10 ASVs identified as significantly different across rumen locations (front, middle, back) by DESeq2 (FDR < 0.05).** Boxplots show the distribution of relative abundances (y-axis) across the three rumen sampling locations, with individual points indicating samples and shapes denoting cows. Horizontal bars and asterisks denote significant pairwise contrasts (FDR corrected p-value < 0.05, ** < 0.01, *** < 0.001). Together, these ASVs accounted for a small fraction of the community (median ~0.6% of total abundance), underscoring that location effects were restricted to low-abundance taxa.

**Supplementary Figure S4.** Predicted functional pathway profiles across rumen sampling locations. Principal coordinate analysis (PCoA) based on Bray-Curtis dissimilarities of PICRUSt2-predicted pathway abundance profiles from rumen fluid samples collected from three rumen locations: Front, Middle, and Back. Points represent individual samples (n = 36), colours indicate sampling location, and shapes indicate cow identity (n = 6). Ellipses show the 80% confidence region for each sampling location. The first two PCoA axes explained 38.8% and 20.8% of the variation, respectively. Predicted pathway profiles showed substantial overlap among rumen locations, and PERMANOVA with permutations restricted within cow detected no significant effect of sampling location (R² = 0.048, p = 0.242). Because pathway profiles were inferred from 16S rRNA gene data, these results should be interpreted as exploratory functional predictions rather than direct metagenomic evidence.
